# Statin-Associated Polymyalgia Rheumatica. An Analysis Using WHO Global Individual Case Safety Database: A Case/Non-Case Approach

**DOI:** 10.1371/journal.pone.0041289

**Published:** 2012-07-23

**Authors:** Hilda J. I. de Jong, Siti R. F. Saldi, Olaf H. Klungel, Rob J. Vandebriel, Patrick C. Souverein, Ronald H. B. Meyboom, J. L. M. (Anneke) Passier, Henk van Loveren, Jan Willem Cohen Tervaert

**Affiliations:** 1 Laboratory for Health Protection Research, National Institute for Public Health and the Environment, Bilthoven, Netherlands; 2 Department of Toxicogenomics, Maastricht University Medical Centre, Maastricht, Netherlands; 3 Division of Pharmacoepidemiology and Clinical Pharmacology, Department of Pharmaceutical Sciences, Faculty of Sciences, Utrecht Institute for Pharmaceutical Sciences, Utrecht University, Utrecht, Netherlands; 4 Uppsala Monitoring Centre, World Health Organisation Collaborating Centre for International Drug Monitoring, Uppsala, Sweden; 5 Netherlands Pharmacovigilance Centre Lareb, ’s-Hertogenbosch, Netherlands; 6 Division of Clinical and Experimental Immunology, Department of Internal Medicine, Maastricht University Medical Centre, Maastricht, Netherlands; University of Michigan, United States of America

## Abstract

**Objective:**

To assess whether there is an association between statin use and the occurrence of polymyalgia rheumatic (PMR) in the spontaneous reporting database of the World Health Organisation (WHO).

**Methods:**

We conducted a case/non-case study based on individual case safety reports (ICSR) in the WHO global ICSR database (VigiBase). Case reports containing the adverse event term polymyalgia rheumatica (WHOART or MedDRA Preferred Term) were defined as cases. Non-cases were all case reports containing other adverse event terms. Each case was matched to five non-cases by age, gender, and time of reporting. Case reports regarding a statin as suspected or concomitant drug were identified using the Anatomical Therapeutic Chemical (ATC) classification. Multivariate logistic regression was used to calculate reporting odds ratios (RORs) with 95% confidence intervals (CI).

**Results:**

We identified 327 reports of PMR as cases and 1635 reports of other ADRs as non-cases. Among cases, statins were more frequently reported as suspected agent (29.4%) compared to non-cases (2.9%). After adjustment for several covariates, statins were significantly associated with reports of PMR (ROR 14.21; 95% CI 9.89–20.85).

**Conclusion:**

The results of this study lends support to previous anecdotal case reports in the literature suggesting that the use of a statin may be associated with the occurrence of PMR. Further studies are needed to study the strength of the association in more detail and to elucidate the underlying mechanism.

## Introduction

The 3-hydroxy-3-methylglutaryl coenzyme A (HMG-CoA) reductase inhibitors, or statins, effectively lower cholesterol levels and significantly reduce the risk of cardiovascular events [1].

Recently, several studies have shown that these agents have anti-inflammatory and immunomodulatory properties which may eventually lead to immune dysregulation [2], [3]. Hence, statins might facilitate the development of autoimmunity, eventually resulting in autoimmune diseases. Previously, we observed in a population-based study that statins were associated with an increased risk of developing RA [4]. Furthermore, cases of statin-associated lupus-like syndrome, dermatomyositis, and vasculitis have been reported [5]–[11] Moreover, in a study comprising data from spontaneous case reports we have found an association between statin use and the occurrence of a lupus-like syndrome [12].

Three case reports suggested that statin use can trigger the development of polymyalgia rheumatica (PMR) [9]–[11]. PMR is an inflammatory rheumatic disease predominantly seen in the elderly and characterised by muscle pain and morning stiffness in the neck, shoulders, and/or pelvic girdle [13], [14]. The association between statin use and PMR has not yet been studied in depth. Therefore, we evaluated the association between statin use and the occurrence of PMR, using a case/non-case approach, in Vigibase the database of the WHO Uppsala Monitoring Centre containing individual case safety reports (ICSRs) of adverse drug reactions (ADRs).

## Methods

### Study Population

The association between the use of statins and PMR was evaluated using the database of the World Health Organisation Uppsala Monitoring Centre (WHO UMC), Sweden. The database (VigiBase) contains the global ICSRs of suspected adverse reactions to pharmaceutical products submitted through National Pharmacovigilance Centres by 90 countries around the world. When we extracted the data for the present study, the database contained more than 4.6 million ICSRs of suspected ADRs [15]. At the national level, ADRs are reported by health-care professionals, pharmaceutical companies, and in some countries by patients. Details about suspected ADRs such as age, gender, reporting date, country, nature of the ADR, suspected drugs, concomitantly used drugs, and interacting drugs are available in the VigiBase. ADRs are coded according to WHO Adverse Reaction Terminology (WHO-ART) or the Medical Dictionary for Regulatory Activities (MedDRA) [15]. The reported drugs are encoded using the WHO Drug Dictionary Enhanced, which includes the WHO Anatomical Therapeutic Chemical (ATC) classification [16]. Information in these reports is not homogenous, at least with regard to origin, completeness of documentation or the likelihood that the suspected drugs caused the adverse events [15].

### Design

A case/non-case approach was used to evaluate the association between the use of statins and PMR. In VigiBase, cases were identified as all ICSRs of ADRs containing the WHO-ART or MedDRA preferred term ‘polymyalgia rheumatica’ [15]. Reports were only included when data on gender and age were available. Since we were interested in incident cases of PMR, ADR-reports with the preferred term ‘polymyalgia rheumatica aggravated’ were excluded from the study. Each case was matched to five non-cases by age, gender, and calendar year of reporting. Non-cases were reports concerning all other adverse reactions.

### Definition of Exposure

Exposure to statins was defined as the reporting of statins as a suspected or concomitant drug for an ADR. The ATC codes for statins were C10AA (HMG-CoA reductase inhibitors), C10BA (HMG-CoA reductase inhibitors in combination with other lipid modifying agents), and C10BX (HMG-CoA reductase inhibitors and other combinations) [16].

### Covariates

Concomitant medication such as drugs with immunomodulatory activity, i.e., use of anti-arrhythmic drugs, antihypertensives, antidiabetic agents, non-steroidal anti-inflammatory drugs (NSAIDs), antidepressants, anti-epileptics, and proton pump inhibitors were considered as covariates [17].

### Statistical Analysis

Characteristics of the cases and non-cases were analysed using T-test, Chi-square test and Fisher’s exact test as appropriate. Means and standard deviations (SD) or percentages were obtained for continuous and categorical variables, respectively. The association between reporting of statins and PMR was assessed using logistic regression analysis and expressed as Reporting Odds Ratios (ROR) accompanied with 95% confidence intervals (CI). The ROR provides a ratio of the odds of exposure in reports of cases and non-cases. The ROR was calculated by dividing the numerator (the number of cases with statins as the suspected drug divided by the number of cases with another suspected drug) by the denominator (the number of non-cases with statins as the suspected drug divided by the number of non-cases with another suspected drug). Covariates that acted as confounders were included in the model if each of them induced a change of crude β estimates of the exposure-outcome association of at least 10% [18]. All tests were two-sided with a rejection of the null hypothesis at a p-value of less than 0.05. All the analyses were conducted using SPSS 16.0 statistical software (SPSS Inc. Chicago, Illinois, USA).

### Sensitivity Analysis

We carried out five sensitivity analyses: 1) since reporters may not always be aware of statin-associated PMR we expanded the definition of statin use by including reports where statins were classified as concomitant (i.e. unsuspected) drug for an ADR.

To explore the influence of potential misclassification of patients with an ADR-report of PMR, we conducted four additional sensitivity analyses: 2) including only ADR-reports reported by physicians, 3) including only ADR-reports of patients older than 50 years [19], [20], 4) defining cases of PMR as an ADR with the single WHO-ART or MedDRA adverse reaction term PMR in which statins were reported as suspected drug, and 5) defining cases of PMR as only ADR of WHO-ART adverse reaction term PMR in which statins were reported as suspected or concomitant drug. In the latter two analyses, ADRs with two or more WHO-ART adverse reaction terms were excluded from the analysis.

## Results

### Baseline Characteristics

**Figure 1:**
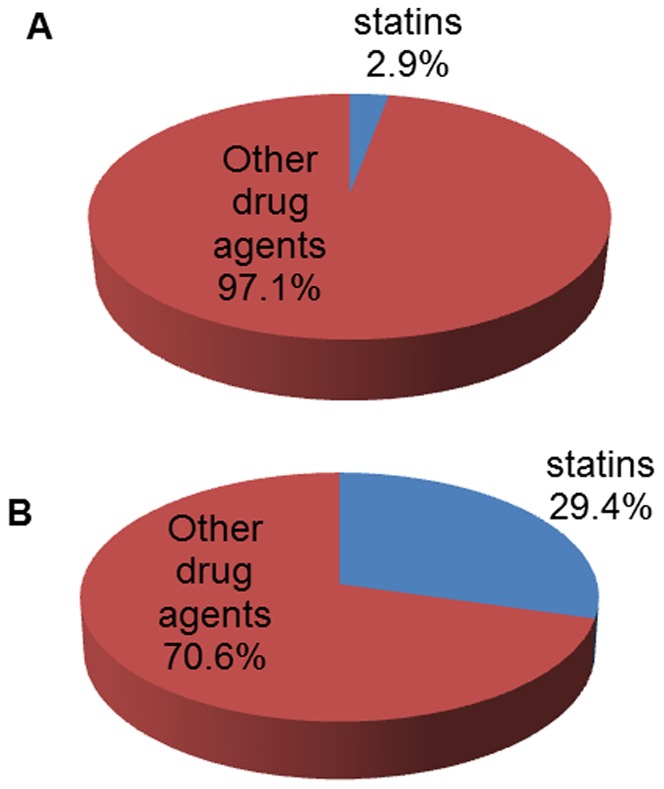
Exposure to statins in cases and non-cases. **1A.** Exposure to drugs in reports of other adverse drug reactions (non-cases). **1B.** Exposure to drugs in reports of polymyalgia rheumatica (cases).

In VigiBase, we identified 327 reports of PMR (cases) that were matched with 1,635 reports of other ADRs (non-cases). The distribution of baseline characteristics for cases of PMR and non-cases are shown in Table 1. Characteristics were similar between cases and non-cases except for anti-depressant and anti-arrhythmic drugs that were more frequently reported in non-cases than in patients with PMR. In 104 of 327 cases of PMR, statins were reported as suspected or concomitant drug. Of these 104 cases, 96 reported statins as suspected drug whereas eight reported statins as concomitant drug. The distribution of statins reported as suspected drug in cases and non-cases are presented in figure 1.

**Table 1:** Baseline characteristics of the Polymyalgia Rheumatica study population.

Characteristics	Cases	Non-cases	p-value
	(n = 327)	(n = 1,635)	
Mean Age (SD), y	67.7 (11.0)	67.7 (11.0)	NA*
Age categories			
<50, % (n)	6.1 (20)	6.1 (100)	NA*
≥50, % (n)	93.9 (307)	93.9 (1,535)	NA*
Sex			
Male, % (n)	37.9 (124)	37.9 (620)	NA*
Female, % (n)	62.1 (203)	62.1 (1,015)	NA*
Statins			
Suspected, % (n)	29.4 (96)	2.9 (47)	<0.001
Suspected or Concomitant, % (n)	31.8 (104)	7.9 (129)	0.08
Comedication			
Anti-arrhythmic drugs, % (n)	2.8 (9)	5.7 (93)	0.03
Antihypertensives, % (n)	26.0 (85)	21.7 (354)	0.09
Antidiabetics, % (n)	4.3 (14)	5.3 (86)	0.46
Non-statins Lipid modifyingagents, % (n)	0.9 (3)	0.7 (12)	0.73
NSAIDs†, % (n)	2.1 (7)	1.5 (24)	0.34
Corticosteroids, % (n)	2.5 (8)	1.5 (25)	0.24
DMARDs‡, % (n)	1.2 (4)	1.8 (30)	0.64
Antidepressants, % (n)	1.2 (4)	4.5 (74)	0.003
Antiepileptics, % (n)	0.3 (1)	1.5 (25)	0.10
Acid inhibitors, % (n)	4.6 (15)	3.6 (59)	0.40

* NA indicate not applicable because cases and non-cases were matched by age and gender.

† NSAIDs: Non-steroidal anti-inflammatory drugs.

‡ DMARDs: Disease-modifying anti-rheumatic drugs.

The characteristics of 96 cases of PMR with reported statins as suspected drug are presented in table 2. Most of these cases were reported by physicians in the late ‘90s and mainly originated from the United States, Germany, and Great Britain. Simvastatin and atorvastatin were more often reported as the suspected drug than other types of statins. The time to onset of PMR after starting statins ranged from one day to 5.7 years (mean, 11.9 months; median, 3.7 months). In 26 of 96 ADR-reports statin withdrawal was recorded. Of these 26 reported cases, eight cases reported that PMR abated while eight cases reported no effect. In six cases, a rechallenge with statins was reported resulting in the recurrence of PMR.

**Table 2:** Detailed information on the 96 case reports with statins as suspected drug.

Characteristic	No. suspected statins	Characteristic	No. suspected statins
	(n = 96)		(n = 96)
**Year of reporting**		**Time to onset (days)**	
1990–1999	59	1–90	35
2000–2006	37	91–365	17
**Type of statin**		>365	23
Simvastatin	35	Not recorded	21
Pravastatin	8	**Outcome**	
Lovastatin	10	Recovered	15
Atorvastatin	26	Not (or not yet) recovered	23
Fluvastatin	6	Recovered with sequelae	3
Cerivastatin	6	Not recorded	55
Rosuvastatin	5	**Dechallenge (Action)**	
**Country of origin**		Drug withdrawn	26
Australia	9	Dose not changed	12
Canada	4	Not recorded	58
Finland	2	**Dechallenge (Result)**	
France	2	Reaction abated	8
Germany	18	No effect observed	8
Great Brittan	15	Not applicable	12
Ireland	1	Not recorded	68
New Zealand	3	**Rechallenge (Action)**	
Norway	1	Rechallenge	6
Sweden	3	No rechallenge	20
Switzerland	1	Not recorded	70
The Netherlands	3	**Rechallenge (Result)**	
United States	34	Reaction recurred	6
**Reporter**		Not applicable	20
General Practitioner	31	Not recorded	70
Physician	8	**Causality**	
Specialist	5	Probable	3
Hospital	2	Possible	21
Manufacturer	2	Not assessed	10
Consumer	2	Not recorded	62
Other*	2		
Not reported	44		

* “Other” includes consumer reports and various types of reports from other health professionals then physicians.

### Association between Reporting of Statins and PMR

The association between the use of statins and PMR is shown in Table 3. Overall, statins were more often reported in patients with PMR in comparison with patients who had experienced other ADRs (adjusted ROR 14.21 [95% CI 9.89–20.85]; P = 0.001). The results were consistent when we conducted five sensitivity analyses; 1) by expanding the exposure definition to statins as suspected or concomitant drug for an ADR, the adjusted ROR for the occurrence of PMR was 6.03 (95% CI 4.40–8.25; P = 0.001), 2) including only ADR-reports reported by physicians: (adjusted ROR 14.70 [95% CI 7.07–30.65]; P = 0.001), 3) including only ADR-reports of patients older than 50 years: (adjusted ROR 14.01 [95% CI 6.98–24.83]; P = 0.001), 4) including only ADR-reports of PMR without other ADRs, in which statins were reported as suspected drug: (adjusted ROR 13.90 [95% CI 8.65–22.35]; P = 0.001), or 5) including only ADR-reports of PMR without other ADRs and in addition where statins were reported as suspected or concomitant drug: (adjusted ROR 5.75 [95% CI 3.74–8.83]; P = 0.001).

**Table 3:** Association between the use of statins and polymyalgia rheumatica (PMR).

Characteristics	Cases (%)	Non-cases (%)	ROR Crude (95% CI)	ROR adjusted (95% CI)||	*p* value¶
	n = 327	n = 1635			
Suspected statins	96 (29.4)	47 (2.9)	14.43 (9.89–21.05)	14.21 (9.69–20.85)	<0.001
Sensitivity analysis 1*	n = 327	n = 1635			
Suspected or concomitant statins	104 (31.8)	129 (7.9)	5.66 (4.20–7.63)	6.03 (4.40–8.25)	<0.001
Sensitivity analysis 2†	n = 76	n = 848			
Suspected statins	18 (23.7)	19 (2.2)	14.15 (6.97–28.75)	14.70 (7.07–30.65)	<0.001
Sensitivity analysis 3‡	n = 307	n = 1535			
Suspected statins	88 (28.6)	43 (2.8)	14.22 (7.15–26.92)	14.01 (6.98–24.83)	<0.001
Sensitivity analysis 4§	n = 157	n = 1635			
Suspected statins	44 (28.0)	47 (2.9)	14.18 (8.93–22.52)	13.90 (8.65–22.35)	<0.001
Sensitivity analysis 5§	n = 157	n = 1635			
Suspected or concomitant statins	46 (29.3)	129 (7.9)	5.55 (3.72–8.29)	5.75 (3.74–8.83)	<0.001

* cases of PMR were defined as all ADR-reports of PMR.

† only ADR-reports reported by physicians.

‡ only ADR-reports of patients older than 50 years.

§ cases of PMR were defined as only a report with the preferred term “PMR”.

|| adjusted for age, gender, reporting year, the use of anti-arrhythmic drugs, antihypertensives, anti-depressants, and anti-epileptics.

¶ *p* values are for ROR adjusted.

## Discussion

In the present study, we observed an association between statin use and reporting of PMR in VigiBase. In six reports the recurrence had been recorded of PMR after reexposure to statins, which strengthened the suspicion regarding the drug’s causal involvement in these patients. In the absence of information on the treatment of the PMR, in our study it could not be determined whether clinical improvement was related to statin discontinuation or to treatment with corticosteroids. In two previously published cases [9], [11], however, information on treatment regimes has been provided. The observation that clinical improvement had occurred within a month after discontinuation of the statin and without the administration of corticosteroids, was highly suggestive of a causal role of the statin in these two patients.

In our study, cases were predominantly older than 50 years and female (67%) which is in line with published studies reporting incidence rates of non-drug associated PMR [21], [22].

Several limitations of our study need to be addressed. First, data were obtained from a spontaneous reporting system without additional clinical assessment or qualitative verifications by the authors. For instance, in many ADR-reports no information was available about the withdrawal of statins, the course of PMR and the response to steroid therapy.

Second, there is gross but variable underreporting and it is likely that only a fraction of the actual adverse events that occurred (perhaps less than 10%) has been reported [23], [24]. ADRs to relatively new drugs, severe ADRs, and ADRs which are not listed in the summary of product characteristics tend to be more often reported [23]. To control for possible time trends of reporting, we matched non-cases for the calendar year of reporting.

Third, we cannot exclude the possibility of unmeasured and/or inadequately measured residual confounding. Furthermore, in VigiBase confounders are difficult to determine because they were not always recorded in the reports. Unfortunately, in our patients no data were available on vitamin D deficiency, which may be an important risk factor for statin-associated muscle complaints [25]. Recently, a case report [26], case series [27], and two cross sectional studies have found an association between vitamin D insufficiency and statin-induced myalgia [28], [29]. Importantly, vitamin D has been shown to modulate the immune response [30]. For instance in humans, high doses vitamin D therapy results in the inhibition of T helper (Th) 1 and Th17 cells and the promotion of Th2 and regulatory T cells [31].

Fourth, reporters may not be aware of the possible association between PMR and the use of statins use, and therefore it may not have been reported, or the statin may have been regarded as a concomitant drug only. In a sensitivity analysis including all reports of statins, suspected or not, the association between statin use and PMR was still observed, although somewhat attenuated.

Fifth, clinical details about the patients with PMR were scarce and we were not able to recognise possible diagnostic misclassification [32]. The reports in Vigibase of PMR were submitted by medical specialists, GPs, manufactures or patients. Information on clinical features, such as elevation of the inflammatory markers (erythrocyte sedimentation rate (ESR) and C-reactive protein (CRP)), response to steroids and disease course, were often not recorded. However, when we only included case reports from physicians, the association between statin use and the occurrence of PMR was still present.

Since PMR occurs almost exclusively in patients aged 50 years and older [19], [20], we performed a sensitivity analysis in which we excluded patients who were younger than 50 years from the study. We still found an association between statin use and the occurrence of PMR.

The symptoms of PMR are very characteristic, although other conditions may mimic PMR [13], [14]. Statins are an established cause of muscular injury and specifically the inclusion of cases of myositis, non-specific myalgias, and/or myopathy may have occurred, the more so since laboratory findings, i.e., serum CK were often not recorded. On the other hand, patients with statin-associated myopathy may have normal serum CK levels which make it sometimes difficult to distinguish PMR from myopathic syndromes [33].

As yet, the possible pathophysiology underlying statin-associated PMR is uncertain.

Recently, it has been postulated that in statin-associated necrotising myopathy, statins may induce neo-antigens as a result of muscle damage which are subsequently presented to the immune system [34], [35]. A similar mechanism may be operative in statin-associated PMR.

We believe that the use of a case/non-case approach in a study with ICSRs of ADRs is, notwithstanding the limitations of our data, an appropriate approach in pharmacovigilance and drug safety research [36]. To our knowledge, this is the first study to assess the association between statin use and the occurrence of PMR in a large spontaneous reporting database. Our findings are consistent in various sensitivity analyses.

We postulate that the use of statins may be associated with an increased occurrence of PMR. Our study presents a pharmacovigilance signal and supports previous anecdotal case reports. We think that further research towards confirming and explaining the association between statin use and PMR is warranted.
